# Physical developmental cues for the maturation of human pluripotent stem cell-derived cardiomyocytes

**DOI:** 10.1186/scrt507

**Published:** 2014-10-20

**Authors:** Renjun Zhu, Adriana Blazeski, Ellen Poon, Kevin D Costa, Leslie Tung, Kenneth R Boheler

**Affiliations:** Department of Biomedical Engineering, The Johns Hopkins University School of Medicine, Baltimore, MD 21205 USA; Stem Cell and Regenerative Medicine Consortium, University of Hong Kong, Hong Kong, SAR China; Icahn School of Medicine at Mount Sinai, Cardiovascular Research Center, New York, NY 10029 USA; Division of Cardiology, The Johns Hopkins University School of Medicine, Baltimore, MD 21205 USA

## Abstract

Human pluripotent stem cell-derived cardiomyocytes (hPSC-CMs) are the most promising source of cardiomyocytes (CMs) for experimental and clinical applications, but their use is largely limited by a structurally and functionally immature phenotype that most closely resembles embryonic or fetal heart cells. The application of physical stimuli to influence hPSC-CMs through mechanical and bioelectrical transduction offers a powerful strategy for promoting more developmentally mature CMs. Here we summarize the major events associated with *in vivo* heart maturation and structural development. We then review the developmental state of *in vitro* derived hPSC-CMs, while focusing on physical (electrical and mechanical) stimuli and contributory (metabolic and hypertrophic) factors that are actively involved in structural and functional adaptations of hPSC-CMs. Finally, we highlight areas for possible future investigation that should provide a better understanding of how physical stimuli may promote *in vitro* development and lead to mechanistic insights. Advances in the use of physical stimuli to promote developmental maturation will be required to overcome current limitations and significantly advance research of hPSC-CMs for cardiac disease modeling, *in vitro* drug screening, cardiotoxicity analysis and therapeutic applications.

## Introduction

Human pluripotent stem cells (hPSCs) of embryonic (embryonic stem cells (ESCs)) or experimental (induced pluripotent stem cells (iPSCs)) origin [1–5] represent the most viable cell source for *in vitro* generation of large numbers of cardiomyocytes (CMs). The directed differentiation of hPSCs to CMs has led to important research advances, including innovative platforms for the study of human development and for disease modeling. It has also reaffirmed the promise of cardiac regenerative medicine with immunologically compatible cells. To date, research has focused justifiably on cellular and molecular mechanisms that control induction, differentiation, proliferation and scalability of CM production [6, 7]. These efforts have led to CM differentiation protocols ranging from monolayer to cell aggregate systems with diverse chemical additives (for example, bone morphogenic protein and activin agonists versus Wnt inhibitors) and a variety of culture techniques (plate, flask, bioreactor) [6, 7] that can be employed for basic cell biology analyses [8, 9], generation of engineered tissue constructs [10–13], and testing of regenerative potential after transplantation in experimental models of heart failure [14].

Despite these advances, a major hurdle for the experimental and clinical use of these cells has been their phenotypic 'immaturity’ *in vitro*. In contrast to native adult CMs, hPSC-derived CMs (hPSC-CMs) are characterized as having small size and low capacitance, absence of T-tubules, lack of well-formed sarcomeres, poor overall calcium storage and handling, absence of multinucleation, relatively low numbers of mitochondria, metabolic dependence on glycolysis, and innate automaticity [15–18]. When transcriptionally compared to native human cardiac muscle cells, hPSC-CMs most closely resemble cells from embryonic or fetal heart [19].

Developmentally, physical cues and environmental factors are paramount for the production of structurally and physiologically mature CMs. *In vivo*, mechanical cues from the cell surface (cell-cell and cell-extracellular matrix (ECM) interactions) are converted into intracellular signals that can lead to phenotypic changes through a process known as mechanotransduction (reviewed in [20–24]). In fact, mechanical stretch and electrical activity are extremely potent biological cues that, in the heart, not only regulate contraction amplitude on a beat-by-beat basis, but also drive mechanical and electrical remodeling of the myocardium during cardiac development, hypertrophy, and disease. Heart tissues and individual myocardial cells experience not only self-generated mechanical force, but also passive and active stretching, all of which can activate mechanotransduction pathways. These physical cues require contact-dependent interactions of cells with ECM, with other cells, as well as with physical features associated with their environment (that is, topography). The fibrous topography is associated with anisotropic alignment of CMs within the heart [25] and gives rise to distinct longitudinal and transverse structures, allowing for directionally distinct pathways of force recognition and transmittance [26, 27]. Indeed, static transverse and longitudinal loading can differentially activate stress-induced mitogen-activated protein kinase (MAPK) [28] and alter cardiac-specific protein secretion [29], while anisotropic mechanosensing with focal adhesion kinase (FAK) phosphorylation has also been reported in neonatal rat ventricular myocytes (NRVMs) [30]. Despite these scientific advances, much less is known about the events that take place in developmentally 'immature hPSC-CMs’.

Here, we review structural components and physical stimuli that may influence hPSC-CM developmental maturation *in vitro*, and discuss data from animals and humans to describe known mechanisms. We focus on mechanical and electrical factors, and touch upon metabolic and hypertrophic signals that have been implicated in the adaptation of hPSC-CMs in two dimensions and, where available, cells engineered in three dimensions. We illustrate how *in vitro* differentiated hPSC-CMs can respond to some of the same physical cues present in embryonic, fetal and adult heart but point out that these factors are preferably interpreted in a three-dimensional context that can be recapitulated *in vitro*. We also highlight areas that are currently not well represented in published works, including the interactions with non-myocytes and application of transcriptomics to electrical and mechanical transduction events in hPSC-CMs. By shedding light on these areas, future research advances may overcome current limitations of hPSC-CMs for reliable disease modeling, drug discovery, cardiotoxicity testing and studies of developmental maturation.

## Heart development and physical cues

Heart development and cell growth involve dynamic interactions between genetic and epigenetic or environmental factors [31] in a spatially complex manner [32]. In response to transcriptional cascades and morphogen gradients, cells in the lateral plate mesoderm and secondary heart fields commit to the CM lineage and presumably acquire an epigenetic phenotype that impedes or prevents de-differentiation. In the embryo and early fetal stages, fully committed CMs increase organ mass mainly through an increase in cell numbers (hyperplasia). During subsequent fetal and perinatal stages, mass increases through hyperplasia and through increased cell size and volume (hypertrophy). Post-natally, increases in cardiac mass occur almost exclusively by hypertrophy. During the perinatal period, hemodynamic loads increase dramatically as the heart assumes its primary role as a circulatory pump. Altered mechanical stimuli include a marked pressure increase and large pulsatile volume changes. In response to the increased work load and energy demand, mitochondrial numbers increase, mitochondrial oxidative metabolism is up-regulated as fatty acids become available, while glycolytic metabolism becomes down-regulated [33]. The major changes in cell phenotype and function - including maturation of the sarcoplasmic reticulum, reversal of the (initially negative) force-frequency relationship [34], multinucleation, and the localization of gap junctions to intercalated discs at cell termini - all occur during the perinatal and early childhood periods. Heart rate, blood pressure, and diastolic stiffness continue to change throughout adolescence and into adulthood and old age. In the adult, the myocardium consists of myofibrils composed of rod-shaped CMs located next to fibroblasts and blood vessels, and these cells are held in place by the ECM and physical interactions with other CMs. The development and maturation of CMs from the fetal to adult stages of life rely on a balance between extrinsic and intrinsic mechanical loads that regulate protein synthesis, sarcomere assembly, cell size, contractile activity, and interactions with other cells and the ECM [35], which together ultimately determine the geometry and pump function of the heart.

Mechanical forces have a continuous and crucial regulatory role in cardiogenesis, cardiac growth, development and maintenance. In the developing mammalian heart, two types of contractile or intrinsic mechanical activity are observed that are believed to involve actin-myosin interactions. The first, which is beyond the scope of this review, is cytoskeletal contraction, a process that drives morphogenesis and cardiac looping [31]. The second is associated with the rhythmic contraction of heart muscle with each cardiac cycle, which subjects CMs to continuous cyclic mechanical strain. Essentially, electrical excitation of CMs is converted into mechanical movement through excitation-contraction coupling, involving regulation of cytosolic calcium and cycling of actomyosin cross-bridges. Individual ventricular CMs experience changes in length and load with each contraction, and cross-bridge interactions are strongly influenced by external signals, like venous return [36]. Through length-dependent (that is, Frank-Starling mechanism) and frequency-dependent contractile properties, contractility can be adjusted on a beat-to-beat basis to accommodate changes in physiologic activity and metabolic demand. When exposed to sustained long-term changes in loading conditions, CMs have the ability to remodel to maintain cardiac performance and restore homeostasis. CMs elongate in response to increased diastolic strain by adding sarcomeres in series, and they thicken in response to elevated systolic stress by adding sarcomeres in parallel. Myocytes do this while maintaining resting sarcomere length close to its optimal value near the peak of the length-tension curve.

Mechanical forces transmitted across the ECM or between cells affect assembly and organization of the ECM, gene transcription, growth, differentiation, apoptosis, signal transduction, electrical coupling and even tissue morphogenesis [37–40]. Although little is known developmentally, mechanical stresses during the cardiac cycle in adult CMs are transmitted through the cytoskeleton and across cell-cell (intercalated discs) and cell-ECM (focal adhesions) complexes to impact the dynamics of physical shortening and tension development. Focal adhesions at the ends of CMs and at costameres aligned with Z-discs couple the ECM to transmembrane integrin receptors. Integrins are cell surface, membrane-spanning receptors that mediate cell-matrix interactions in all higher organisms and are linked to the actin cytoskeleton via adaptor proteins like vinculin, paxillin and α-actinin [39]. These membrane proteins utilize a variety of downstream kinases to regulate signals within the cells. The major signal transduction molecule involves FAK, which can regulate pathways involved in transcriptional control, cell remodeling [41] and response to cardiac hypertrophy [42].

Intercalated discs (ICDs) are highly organized intercellular adhesion structures composed of fascia adherens (adherens junctions), macula adherens (desmosomes) and gap junctions. Fascia adherens and desmosomes are necessary for mechanically coupling and reinforcing CMs [43]. Fascia adherens are the primary force-transmitting structures and are anchoring sites to the actin cytoskeleton. They are composed of cadherins (N-cadherin), which are responsible for Ca^2+^-dependent homophilic cell-cell adhesion, catenin-related proteins (vinculin and α-actin) that link the ICD to the cytoskeleton, and cytoplasmic catenins (α-, β-, γ-catenin (plakoglobin)) that bind cadherins and regulate their adhesive activity. α-Catenins are thought to directly link the cytoplasmic domain of cadherin to the actin cytoskeleton. Fascia adherens play critical roles in cardiac development, disease and in arrhythmias [43]. Desmosomes are also involved with force transmission between CMs and play an important role to resist shearing forces, which can arise from the laminar architecture of myocardium [44, 45], and can influence ion channel trafficking to the CM membrane [46]. Desmosomes contain desmosomal cadherins (desmocollin, desmoglein), which bind to the armadillo family proteins (junctional plakoglobin, plakopilin), which, in turn, anchor to a plakin family member (for example, desmoplakin) that connects to the intermediate filament cytoskeleton [47]. Over-expression of N-cadherins in mouse models causes dilated cardiomyopathies, while desmosome mutations in human lead to arrhythmogenic right ventricular dysplasia/cardiomyopathy (ARVD/C) and impaired mechanical coupling between individual cells with possible impairment of electrical coupling [48, 49]. Morphologically, ICDs are normally arranged at the ends of adult CMs, but in immature or diseased cells the adherens junctions and gap junctions can be located on the lateral sides of CMs.

Gap junctions are necessary for rapid electrical transmission between cells [43]. They are composed of six connexin molecules and form two half-channels across an intercellular space. When connected, these junctions provide a pathway for the passage of ions and small molecules (<1,000 Da) between cells [50]. Connexin 43 (Cx43) and N-cadherin share a temporal relationship both in expression and co-localization [39, 51], and the assembly of gap junction channels is preceded by the formation of fascia adherens [52, 53]. During postnatal development, both Cx43 and N-cadherin are distributed in human ventricular cells over the entire surface of the cell. These molecules gradually redistribute to ICDs at the longitudinal ends of the cell, reaching the adult pattern at around 6 years of age [54], although a recent study found that N-cadherin redistributes much more quickly, by around 1 year of age [55]. Gene mutations in connexins have rarely been found to be a cause for human cardiac disease; however, remodeling of connexin isoform expression and changes in gap junction organization are typical traits of ischemic heart disease and failure [50].

**Figure 1:**
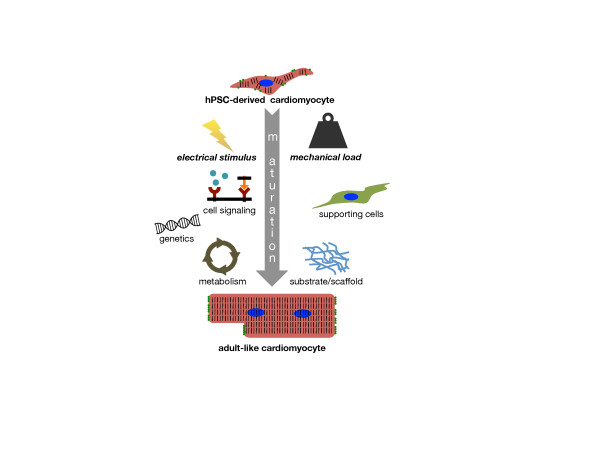
**Schematic diagram illustrating developmental factors that potentially impact the** ***in vitro*** **maturation process from human pluripotent stem cell (hPSC)-derived cardiomyocytes to an adult-like phenotype with highly organized sarcomeres and intercellular junctions.** This review focuses on physical developmental cues from electrical stimulation and mechanical loading, and also mentions factors including genetics, supporting cells and substrate, metabolism, and both circulating and membrane bound signaling molecules.

Given the complexity of mechanical and structural interactions just described in normal development as well as genetic factors (Figure 1), it is challenging to isolate specific mechanical signals that stimulate remodeling responses, particularly since stress and strain (deformation) often co-vary *in vivo* and *in vitro*. External forces from either passive or active wall stress in the heart can increase resting cell length (if during diastole), resist cell shortening (if during systole and less than the cellular contractile force), and paradoxically lengthen the cell (if during systole and greater than the contractile force). Interestingly, significant CM shape change and rearrangement of sarcomeres has been observed *in vitro* using isolated rodent papillary muscles in a controlled muscle culture system [56] even in the presence of the cross-bridge inhibitor 2,3-butanedione monoxime (BDM), which diminishes systolic force. A lack of high shear stress from intracardiac flow leads to abnormal heart development in zebrafish embryos, indicating mechanical load can also play an epigenetic regulating role [57]. Thus, a full understanding of how mechanical and electrical forces may influence hPSC-CM developmental maturation is a challenging proposition, but one that should be amenable to *in vitro* analyses designed to unravel cell autonomous responses versus those that are manifested in response to physical stimuli in two or three dimensions.

## State of hPSC-CMs during differentiation

### Experimental considerations

**Figure 2:**
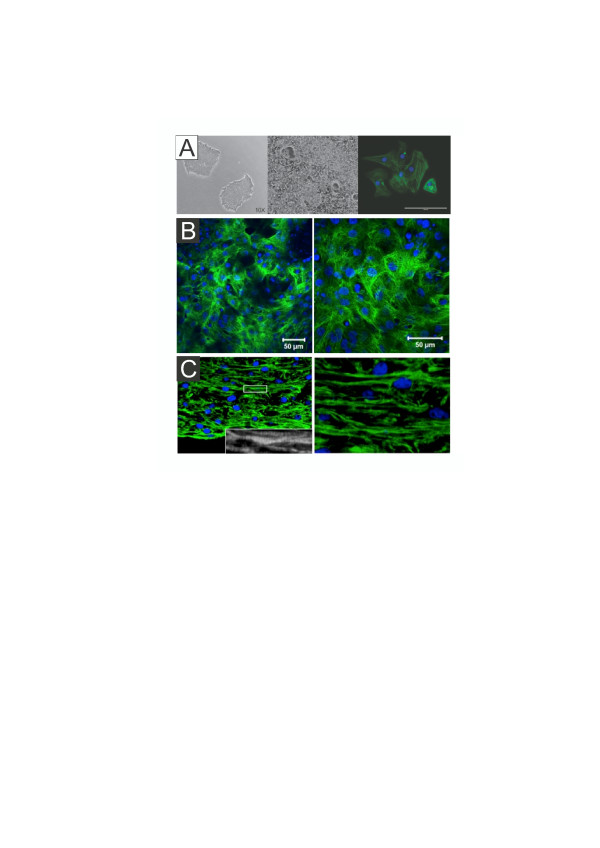
**Representative images of hPSC and hPSC-CM. (A)** Representative images of human pluripotent stem cells (hPSCs) (left), a monolayer culture of hPSC-derived cardiomyocytes (hPSC-CMs; unstained, middle), and dissociated and re-plated human embryonic stem cell-derived cardiomyocytes immunostained with antibodies against cardiac troponin T (TNNT2; right) [139]. **(B)** Cardiac troponin I (TNNI3) immunostaining of a monolayer culture of human induced pluripotent stem cell-derived cardiomyocytes at day 29 of differentiation showing random patterns of striations. **(C)** Immunostaining of a three-dimensional tissue strip with well-aligned troponin-stained hPSC-CMs. Green, TNNT2 labeling **(A, C)**, TNNI3 labeling **(B)**; blue, DAPI labeling.

Relative to adult heart, hPSC-CMs display a developmentally immature phenotype *in vitro*. The resulting phenotype is not, however, constant as the differentiation protocol, time of differentiation, presence of growth factors and ancillary cells, as well as cultivation in two dimensions versus three dimensions all influence the *in vitro* phenotype. Structurally, some of these differences can be visualized by immunostaining with antibodies against sarcomeric proteins like cardiac troponin T (TNNT2) and I (TNNI3) (Figure 2). Under standard two-dimensional conditions, the cardiac troponin arrangements are random, while those in three-dimensional tissue strips are much more aligned. Problematically, published reports on physical cues that affect hPSC-CM structure and function have not taken variables associated with *in vitro* differentiation into account. In fact, data from hPSC-CMs have been obtained with divergent methods ranging from highly efficient to inefficient differentiation protocols that involve monolayers to cell aggregates known as embryoid bodies (EBs) or cardiospheres (Table 1). While most of the published data have employed suspension EBs for generation of hPSC-CMs, the time of cultivation and dissociation protocols from suspension EBs have varied widely. Moreover, when considering physical cues, it is crucial to consider mechanisms that generate force as well as those mechanisms that transmit and coordinate forces within complex tissues. This process involves direct cell-cell interactions through fascia adherens and desmosomes, cell-ECM interactions through focal adhesions, cellular electrical coupling through gap junctions, and signal pathway and transcription factor activation in a two-dimensional and three-dimensional context.

**Table 1:** **Summary of methods and relative maturation states of** ***in vitro*** **derived cardiomyocytes**

Study	Method of differentiation	Maturation state achieved and finding
**Boheler** ***et al*** **. 2014** [139]	Monolayer culture	High purity hESC- and hiPSC-derived CMs
**Cao** ***et al*** **. 2008** [59]	Suspension EB	Expression level of genes encoding structural and force-generating proteins was comparable to fetal heart CMs
**Caspi** ***et al*** **. 2009** [74]	Suspension EB	Presence of functional gap junctions
**Chan** ***et al*** **. 2013** [102]	Suspension EB	Electrical field stimulation increased expression of cardiac-specific genes
		Stimulation promoted a ventricular-like phenotype
		Stimulation improved calcium handling
**Chung** ***et al*** **. 2007** [65]	Suspension EB	Mitochondrial oxidative metabolism is required for differentiation into a functional cardiac phenotype
**Foldes** ***et al*** **. 2011** [116]	Suspension EB	Showed active role for protein kinase signaling in hESC-CM growth and hypertrophy
**Gherghiceanu** ***et al*** **. 2011** [58]	Suspension EB	Ultrastructural features of early and immature phenotype:
		Myofibrils with sarcomeric pattern
		Large glycogen deposits,
		Lipid droplets
		Ca^2+^ release units detected on the sarcoplasmic reticulum
		Underdeveloped intercalated disks
		Spatial location of cells within EBs can affect their phenotype
		Cx43 expression not detected
**Kamakura** ***et al*** **. 2013** [62]	Suspension EB	Including long-term culture (up to 180 days)
		Myofibrils became tightly packed and formed parallel arrays
		Appearance of mature Z-, A-, H-, and I-bands
		M-bands detected in 360-day-old EBs
**Kehat** ***et al*** **. 2004** [75]	Suspension EB	hESC-CM tissue engraftment
		Structural and electromechanical connections with NRVMs
		Rate-responsive biological pacemaker
**Kensah** ***et al*** **. 2013** [99]	Cardiac bodies	Formation of bioartificial cardiac tissue and use of defined animal-free matrix
**Kim** ***et al*** **. 2013** [83]	Suspension EB	Induction of adult-like metabolism is critical for establishing disease onset in patient-specific iPSCs
**Kim** ***et al*** **. 2010** [130]	Suspension EB	Co-culture with non-cardiomyocytes rescued the arrest of electrophysiological maturation observed following hESC-CM isolation from EBs in early cultures
	Purified CMs with a-MHC-Pac^R^	
**Lundy** ***et al*** **. 2013** [60]	Monolayer-based direct differentiation	Late stage (about 100 days) cells exhibit
		organized, longer sarcomeres with aligned Z-disks and organized A- and I-bands
		Dense and aligned myofibrils
		Higher degree of multinucleation
		MYH6 and MYH7 expression level comparable to adult human heart
		Improved contraction, Ca^2+^ handling and AP characteristics
**Matsa** ***et al*** **. 2014** [133]	Suspension EB	Allele-specific RNA interference can rescue diseased phenotype in LQTS cardiomyocytes
**Moore** ***et al*** **. 2008** [71]	Suspension EB	Cx43 mediates the expression of genes involved in cardiogenesis
**Ou** ***et al*** **. 2011** [63]	Hanging-drop EB	Three-dimensional culture and cardiac fibroblast co-culture improved sarcomere organization and Cx43 expression
**Pal** ***et al*** **. 2013** [64]	Suspension EB and monolayer-based direct differentiation	Three-dimensional culture increased the expression level of TNNT2
**Pekkanen-Mattila** ***et al*** **. 2010** [72]	Suspension EB,	Sparse, irregularly distributed Cx43 expression
	END-2 co-culture	
**Poon** ***et al*** **. 2013** [19]	Directed differentiation (suspension EB)	Expression level of structural proteins are lower than in fetal ventricular CMs
	Purified ventricular phenotype	
**Sartiani** ***et al*** **. 2007** [61]	Suspension EB, dissected CMs	Electrophysiological characterization over a 3-month period
		Maturation approaching an adult phenotype
**Thavandiran** ***et al*** **. 2013** [101]	Suspension EB	iPSC-derived engineered cardiac tissue
		Combination of a matrix-based microenvironment, uniaxial mechanical stress and a mixture of cells improved engineered cardiac tissue performance
**Turnbull** ***et al*** **. 2014** [13]	Suspension EB	Expression of cardiac genes approached levels in adult LV myocardium in engineered cardiac tissues
**Xue** ***et al*** **. 2005** [76]	Suspension EB	Functional integration into myocardium
		Cell-based pacemaker
**Zhang** ***et al*** **. 2013** [12]	Suspension EB	Well-developed sarcomeric structures found in three-dimensional cardiac patches
		Upregulated E-C coupling and contractile genes
**Zwi** ***et al*** **. 2009** [73]	Suspension EB	Sparse, irregularly distributed Cx43 expression

AP, action potential; CM, cardiomyocyte; Cx43, connexin 43; EB, embryoid body; E-C, excitation-contraction; hESC, human embryonic stem cell; hESC-CM, human embryonic stem cell-derived cardiomyocyte; hiPSC, human induced pluripotent stem cell; iPSC, induced pluripotent stem cell; LQTS, long QT syndrome; LV, left ventricular; NRVM, neonatal rat ventricular myocyte; TNNT2, cardiac troponin T.

### Structural organization

Relative to adult heart cells, both human ESC-derived CMs (hESC-CMs) and human iPSC-derived CMs (hiPSC-CMs) are characterized by variable degrees of myofibrillar organization, abundant glycogen, and underdeveloped ICDs, all of which contribute to a developmentally immature phenotype [18, 58]. While ultrastructural maturation is analogous for both hESC-CMs and hiPSC-CMs, spatial constraints can also affect their phenotype. In both hESC and hiPSC EBs, cells on the periphery of the EB (small round-shaped three-dimensional structure) are more elongated, rod-shaped, have more oval nuclei, and often have clear cross-striations, while cells in the center are more densely packed, rounded, have more irregularly shaped nuclei, and often have no visible striations [58]. One study, comparing hESC-CMs to fetal heart CMs, found that expression of genes encoding structural and force generating proteins was comparable [59]. Our own transcriptomic study, however, indicated that structural protein transcripts are frequently much higher in fetal ventricular CMs than in hESC ventricular CMs [19], suggesting that additional stimuli are needed to produce more transcriptionally active CMs.

Ultrastructural and functional maturation proceeds during prolonged culture [60, 61]. While early-stage (approximately 30 days of differentiation) hESC-CMs lack sarcomeric elements and exhibit disorganized and sparse myofibrils, late stage (approximately 100 days of differentiation) hESC-CMs and hiPSC-CMs can exhibit organized, longer sarcomeres with clearly aligned Z-discs and organized A- and I-bands, dense and aligned myofibrils, and a much higher degree of multinucleation. M-bands are detected at an even later stage (360 days of differentiation) in hiPSC-CMs [62]. Late stage hPSC-CMs exhibit up-regulation of cardiac structural genes encoding α- and β-myosin heavy chain (MYH6 and MYH7), reaching levels comparable to those found in the adult human heart [60]. Maturation is also evident at a functional level, with late-stage hPSC-CMs exhibiting improved contraction (higher magnitude and slower kinetics) compared to early stage hPSC-CMs [60]. Three-dimensional culture has also been shown to increase the organization of sarcomeric myofilaments [63] and the level of TNNT2 [64] in hESC-CMs. In three-dimensional human engineered cardiac tissues, expression of cardiac genes approach levels in adult left ventricular myocardium with increased time in culture [13]. Transcripts encoding contractile and Ca^2+^ handling proteins like MYH6, MYH7, TNNT2, L-type Ca^2+^ channel, ryanodine receptor, SERCA2a and CASQ2 are up-regulated in three-dimensional constructs versus two-dimensional cultures [10, 12]. Furthermore, hESC-CMs in three-dimensional cardiac patches fabricated with a hydrogel consisting of Matrigel and fibrinogen exhibit well developed sarcomeric structures, as evidenced by α-actinin and TNNT2 striations, longer sarcomeres than in two-dimensional monolayers, and up-regulated excitation-contraction coupling and contractile function genes [12]. However, when compared to spontaneously formed human EBs, MYH6 levels did not differ in three-dimensional engineered heart tissues (EHTs) [11].

Rodent and human membrane structures and organelles, like mitochondria and sarcoplasmic reticulum, undergo developmental changes during differentiation *in vitro*. Ca^2+^ release units, most likely involving ryanodine receptor isoforms, have been detected on the sarcoplasmic reticulum [58], while cationic ion channels on the sarcolemmal membrane exhibit expressional and functional differences as a function of differentiation time [61]. At a functional level, late-stage hPSC-CMs exhibit improved calcium handling (faster calcium transient upstroke and decay) and action potential characteristics (slower spontaneous rate, faster maximum upstroke velocity, larger amplitude, and hyperpolarized mean diastolic potential) than early-stage hPSC-CMs [60]. In the undifferentiated state, mouse ESC (mESC) mitochondria are spherical and exhibit under-developed cristae, while those in mESC-CMs are organized in extended, aligned networks and are rich in cristae [65]. Mitochondrial development during the early differentiation process progresses from random, perinuclear localization to transcellular arrangement. These changes occur in parallel with the development of the contractile apparatus [65]. In humans, mitochondria have similar morphology and distribution in hESC-CMs and hiPSC-CMs derived from human follicle keratinocytes. While mitochondria in hESC-CMs and hiPSC-CMs are closely associated with the sarcoplasmic reticulum, contacts tethering the two organelles are rarely found [58]. Mitochondria thus appear to undergo structural developments with *in vitro* differentiation, but the functional significance of these changes is poorly understood. The influence of mechanical stimulation on these structures and their function is unknown.

The application of three-dimensional cultures can further accelerate functional and organelle maturation of PSC-CMs relative to those found in two-dimensional cultures. Lundy and colleagues [60] found that it took 100 days for hESC-CMs in two dimensions to exhibit more advanced states of developmental maturation. In comparison, mESC-CMs grown as a suspension of cells in a three-dimensional hydrogel, consisting of Matrigel and fibrinogen and having an advanced structural design, achieved significant maturation within as little as 3 weeks. Cells within these three-dimensional patches were aligned with abundant adherens and gap junctions, were highly differentiated, and had fast anisotropic electrical conduction and strong contractile forces [66]. The same patch structure also resulted in advanced functional maturation of hiPSC-CMs [67] and hESC-CMs [12]. Thus, PSC-CMs, when incorporated into three-dimensional tissue engineered constructs, are capable of forming functional tissues with enhanced maturation characteristics [67], and apparently more rapidly than those cultivated in two-dimensional systems.

### Electrical and mechanical junctions

Altered connexin expression in undifferentiated PSCs can affect stem cell properties and differentiation to CMs; however, the results have not been consistent. In the undifferentiated state, Cx43 expression and functional gap junctions are present [68, 69], but down-regulation of Cx43 using small interfering RNA can lead to a decrease in some stemness attributes [70]. Cx43-mediated interactions, however, may not have any impact on stemness of hESCs, since intercellular communication of hESCs with Cx43-down-regulated human adipose-derived stem cells had no effect on selected properties of stemness [70]. Lentivirus-mediated over-expression of Cx43 in hESCs impairs the development of functional CMs in differentiated EBs [71]. Spontaneous beating and expression of mesodermal markers are absent in Cx43-enhanced EBs and, while Cx43-EBs express a variety of gene transcripts associated with terminal cardiac differentiation, the expression of TNNI3 and MLC2v is delayed compared with control EBs. Further, a range of genes affecting cellular growth and proliferation, movement, differentiation, and maintenance are differentially expressed in hESCs with over-expressed Cx43, pointing to the role of Cx43 in both the maintenance of stem cell properties and the regulation of cardiomyogenesis [70, 71].

In hPSC-CMs, Cx43 may vary spatially and functionally with time of differentiation and with culture conditions [72, 73]. Visualized by immunostaining, these junctions often appear sparse and irregularly distributed at the cell membrane, analogous to what is seen in mammalian development. In one study, junctions were undetectable by electron microscopy [58], but in another study, application of a gap junction uncoupler, 1-heptanol, resulted in dose-dependent conduction slowing, suggesting the presence of functional gap junctions in hESC-CMs [74]. Consistent with this assumption, Cx43 mRNA can be detected early in hESC-CM differentiation. Its expression, however, may depend on cultivation conditions, as transcript abundance is enhanced by co-culture with murine embryonic fibroblasts [63]. Three-dimensional culture in a collagen matrix combined with co-culture, which physically affects the local environment, further enhances Cx43 expression at a later stage of differentiation [63]. In advanced cardiac patch structures, Cx43 is present in intercellular gap junctions, but culture of hESC-CMs as a patch does not increase the expression of the Cx43 gene when compared to monolayer culture [12]. Additionally, beating hESC-CMs are capable of pacing NRVM monolayers in co-culture where Cx43 is expressed along the surface of contact between the two cell types [75, 76]. Finally, mESC-CMs are connected to each other by 'nascent ICDs’ composed of fascia adherens and gap junctions [77, 78], and Lucifer yellow spreads to adjacent cells. Electrical propagation across these cells implies that PSC-CMs have some form of functional electrical coupling.

Other than Cx43, an analysis of junctional complex components and their relation to force generation in hPSC-CMs is currently very limited. One study in mESCs showed that FAK is a key regulator of cardiogenesis that helps direct stem cell lineage commitment [79]. Another study of hPSC-CM ultrastructure revealed the presence of desmosomes and fascia adherens, but the typical stepladder pattern characterizing ICDs was not observed [37]. hiPSC- and hESC-CMs were, however, connected by 'primitive ICDs’ [58], which may contribute to the immature electrical properties of these cells. Unlike Cx43, over-expression of N-cadherin [80] in mESCs does not interfere with the formation of functional CMs. In fact, mESC-CMs that over-express N-cadherin show increased levels of Cx43 [80], supporting the idea that adherens junction formation drives connexin expression [81]. N-cadherin has also been used as a surface marker to identify human mesenchymal stem cells that reportedly have increased cardiomyogenic differentiation ability [82]. iPSCs with mutations in desmosome proteins differentiated into CMs exhibit calcium-handling deficits and can be metabolically modulated to recapitulate adult ARVD/C pathologies [83].

## Mechanical and electrical stimulation of hPSC-CMs

### Experimental considerations

Insights into mechanisms underlying the adaptive responses of cardiac cells to external forces have been gained from *in vitro* studies of isolated cells, using precisely controlled timing, magnitude, and direction of the mechanical stimuli [29, 84–87] (Table 2). Substrate stiffness [88, 89] or applied cyclic stretching can significantly impact size, elongation, alignment, protein synthesis and contractile function of cultured CMs [90]. However, experiments such as these are not without significant limitations. Many studies have been performed on cultured neonatal rodent CMs using traditional two-dimensional systems where CMs are grown on planar substrates having supra-physiologic stiffness. This can shield cell-cell junctions from mechanical stress during active contraction and passive relaxation. Two-dimensional structures typically fail to recapitulate important aspects of the natural three-dimensional, anisotropic cardiac mechano-environment that fundamentally impact the cell biology [41, 91]. When purified hESC-CMs have been plated on extracellular matrix components layered on top of a two-dimensional micropatterned design, highly aligned cell aggregates with improved sarcomere structures readily formed [92]; but these types of studies lack an essential component of the myocardium - the cardiac fibroblast [93]. By number, fibroblasts comprise approximately 50% or more of cardiac cells and are a major source of ECM production [94, 95], the composition of which is highly specialized in heart. Moreover, the lack of aligned three-dimensional cell attachments and normal electrical coupling in an appropriate topography and environment is likely to adversely influence tension development and other physiological traits.

**Table 2:** **Effects of external factors on maturation**

External factors	Effects on developmental maturation	Reference
**Substrate stiffness/**	Affects differentiation efficiencies. Intermediate-stiffness hydrogels lead to the highest efficiencies	Hazeltine *et al*. 2014 [97]
**Two/three-dimensional culture**	Increases organization of sarcomeric myofilaments	Ou *et al*. 2011 [63]
		Zhang *et al*. 2013 [12]
	Increases cardiac gene expression	Pal *et al*. 2013 [64]
		Turnbull *et al*. 2014 [13]
	Increases contractile and Ca^2+^ handling protein expression	Tulloch *et al*. 2011 [10]
		Zhang *et al*. 2013 [12]
	Promotes alignment and anisotropy	Liau *et al*. 2011 [66]
	Promotes functional maturation in general	Christoforou *et al*. 2013 [67]
	Two-dimensional alignment and groove widths between 30 and 80 μm promote alignment and improve sarcomere structures	Salick *et al*. 2014 [92]
**Mechanical stimulation**	Increases expression of cardiac α-actin and MYH6, and enhances expression of cardiac transcription factors	Gwak *et al*. 2008 [98]
	Improves tissue morphology and enhances active force levels	Kensah *et al*. 2013 [99]
	Increases cell alignment	Tulloch *et al*. 2011 [10]
		Schaaf *et al*. 2011 [11]
		Thavandiran *et al*. 2013 [101]
		Zhang *et al*. 2013 [12]
	Increases proliferation rates	Tulloch *et al*. 2011 [10]
	Increases AP duration and upstroke velocity, but leads to a less negative MDP	Schaaf *et al*. 2011 [11]
	Increases cell size, cytoskeletal assembly and sarcomeric organization	Foldes *et al*. 2011 [116]
	Cyclic stretch improves TNNT2 and Cx43 expression, increases contraction rates and shortens calcium transients	Mihic *et al*. 2014 [100]
**Electrical stimulation**	Leads to better structured and organized myofilaments	Lieu *et al*. 2013 [15]
	Produces cell elongation, affects expression of a group of cardiac-related genes	Chan *et al*. 2013 [102]
		Chen *et al*. 2009 [104]
	Improves cardiomyocyte alignment, cross-striation patterns and force development	Hirt *et al*. 2014 [103]
**Energy substrate**	Elicits ARVD/C phenotype of increased apoptosis, elevated lipogenesis, and impaired calcium handling in *PKP2* mutants	Kim *et al*. 2013 [83]
	Galactose and fatty acids increase oxidative phosphorylation levels, reserve capacity, and maximum respiratory capacity in mitochondria	Rana *et al*. 2012 [120]
	Glucose depletion along with lactose supplementation increase cardiomyocyte purity	Tohyama *et al*. 2013 [121]
	Induction of mitochondrial biogenesis increases cardiomyocyte differentiation	Prowse *et al*. 2012 [126]
**Other**	Stimulating p38-MAPK increases cell size, improves sarcomere and cytoskeletal assembly	Foldes *et al*. 2011 [116]
		Heineke and Molkentin 2006 [117]
	Thyroid hormone increases cardiomyocyte size, sarcomere length, contractile force and anisotropy	Yang *et al*. 2014 [18]
	Adrenergic agonists produce hypertrophy	Foldes *et al*. 2011 [116]
	IGF1 together with electrical or electromechanical stimulation improve NRVM engineered tissue function, SERCA2a and TNNT2 expression	Park *et al*. 2014 [119]
		Morgan and Black 2014 [118]

AP, action potential; ARVD/C, arrhythmogenic right ventricular dysplasia/cardiomyopathy; Cx43, connexin 43; IGF-1, insulin-like growth factor 1; MAPK, mitogen-activated protein kinase; MDP, maximal diastolic potential; NRVM, neonatal rat ventricular myocyte; TNNT2, cardiac troponin T.

**Figure 3:**
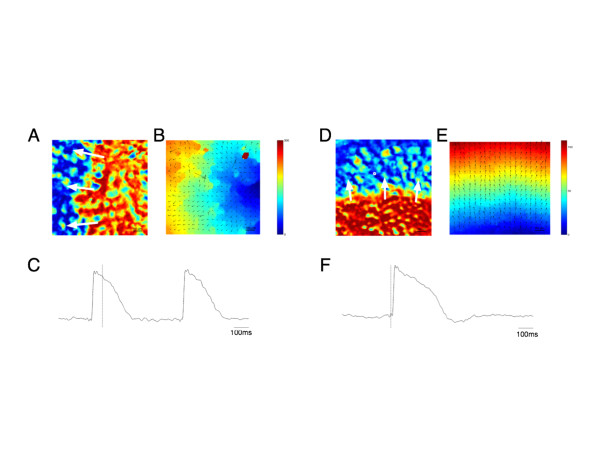
**Optical characterization of hPSC-CM electrophysiology.** Optical mapping of enzymatically digested and re-plated human induced pluripotent stem cell-derived cardiomyocyte monolayers recorded 9 days **(A-C)** and 13 days **(D-F)** after re-plating. **(A)** Transmembrane voltage map of 9-day re-plated monolayer. **(B)** Activation time map and local conduction velocity vectors of **(A)**. **(C)** Action potential recorded from location indicated by white box in **(A)**. Electrical coupling among the cells of this 9-day old monolayer is poor, as indicated by the disorganized activation time map, rough wavefront of the propagating AP and slow conduction velocity (5.4 cm/second). **(D)** Transmembrane voltage map of a 13-day re-plated monolayer. **(E)** Activation time map and local conduction velocity vectors of **(D)**. **(F)** Action potential recorded from location indicated by white box in **(D)**. Electrical coupling was much improved with increased time in culture, as indicated by the near planar propagating action potential as well as faster conduction velocity (10.5 cm/second). Dashed lines in **(C)** and **(F)** indicate the time points of the corresponding transmembrane voltage maps in **(A)** and **(D)**.

To illustrate limitations associated with normal electrical coupling, it is necessary to consider that all single cell studies as well as most two-dimensional and three-dimensional culture systems usually require enzymatic disaggregation. When re-plated or allowed to form tissue-like structures, the plating substrate, cell density and timing of experimentation are critical variables. At very low densities, cells are generally not in direct contact, but retain ion channel activities, which can be measured electrophysiologically. For whole cell patch clamp studies, this is the ideal study design. When intermediate cell plating densities are used, cell-cell contacts form, but a highly coupled syncytium of hPSC-CMs does not. When cells are dissociated and re-plated at a relatively high density to promote syncytium formation, spontaneous electrical activity monitored by optical mapping initially arises only in localized areas. The propagation of these electrical activities is highly disorganized across the monolayer (Figure 3) and is characterized by a slow conduction velocity (Figure 3A-C). This delay may indicate inappropriate gap junction formation. With time, cells show improved electrical coupling, with a better organized propagating wavefront and a much higher conduction velocity (Figure 3D-E). In fact, conduction velocities may continue to increase over a period of 1 month, indicative of a long-term reaction to autonomous electrical activity [96]. Thus, enzymatically digested cells require sufficient time to fully re-establish normal electrical coupling, and the initial lack of directional electrical coupling in high density two-dimensional, and by extrapolation three-dimensional, cultures would be expected to adversely affect contractile force production and downstream mechanosignaling.

### Mechanical stimulation

Spontaneously contracting hPSC-CMs are usually plated on ECM protein-coated tissue culture surfaces, which can impact cardiac differentiation efficiency. The effect of substrate rigidity on this process was demonstrated by culturing hESCs on polyacrylamide hydrogels of different stiffness spanning the physiological range. Substrates with intermediate-stiffness hydrogels led to the highest differentiation efficiencies [97]. This substrate effect depended on the developmental stage of cardiac differentiation, as cells associated with later stages of cardiac specification (that is, mesodermal progenitors) had an apparent loss of substrate sensitivity when compared to hESCs [97].

In contrast to conventional two-dimensional culture of hPSC-CMs on stiff tissue culture surfaces or in suspension as spontaneously formed EBs, three-dimensional culture systems provide a biomimetic environment with controlled biological complexity that can yield valuable insights into the roles of specific physical and mechanical stimuli in the development of CMs. By applying external mechanical stress to the entire three-dimensional construct through custom-made or commercially available loading systems or by varying the matrix composition (and thereby its stiffness), it is possible to control both the static and dynamic load experienced by hPSC-CMs. mESC-CMs under continuous stretch, for example, show increased expression of cardiac α-actin and MYH6 and enhanced expression of transcription factors like Gata4 and Nkx2.5. These stretched cells reportedly form better cell-cell connections that facilitate synchronous contraction both in culture and after implantation onto infarcted rat hearts [98]. Differentiated CMs can also change their structure to align with an applied mechanical stress. Increasing stepwise stretch of mouse iPSC-CM tissue constructs improved tissue morphology (enhanced cellular alignment and sarcomere organization), produced longer sarcomeres, and enhanced active force levels [99]. In hESC- and hiPSC-CM constructs, both static and cyclic uniaxial stress increased cell and matrix fiber alignment, although not to the level observed in adult rat heart [10]. A similar response was observed in an hESC-CM EHT model, where the constructs were subject to mechanical loads produced by their spontaneous contraction. The CMs showed predominantly longitudinal orientation along force lines [11]. In a separate study, cyclical stretch of hESC-CMs seeded onto scaffolds had a greater proportion of TNNT2-positive cells relative to non-stretched controls [100]. The stretched cells were elongated, and demonstrated increased Cx43 expression and faster contraction rates with shorter calcium transient times. In addition to engineered tissue strips, hPSC-CMs in engineered tissue patches also displayed highly aligned CMs in response to stress. Elongated and oriented cells have been observed within the cardiac patches at locations where high uniaxial stress is expected [101]. Notably, the uniformity of CM alignment can be improved by locally controlling the direction of passive tension within the cardiac patch [12].

Mechanical load influences proliferation and sarcomeric organization of hPSC-CMs. Proliferation rates of CMs increased by 21% in cardiac constructs conditioned by cyclic uniaxial stretch relative to unconditioned constructs, and a further increase in proliferation was observed by addition of non-myocyte (endothelial or stromal) supporting cells [10]. Sarcomere organization in stress-conditioned cardiac constructs exhibits cross-striated patterns, similar to native tissue. Contractility of these tissue constructs also benefits from the application of mechanical stimuli, and the tissues show positive inotropic responses to beta-adrenergic stimulation [10, 12]. It is likely that these results can be attributed, at least in part, to the influence of the three-dimensional environment on the cells. In summary, more organized sarcomere structures, increased transcriptional expression of contractile proteins and improved contractility suggest a more mature CM phenotype in mechanically conditioned EHTs.

The presence of passive or active mechanical stimuli improves the electrophysiological function of hPSC-CMs. Microelectrode recordings of cells isolated from EHTs showed an increase in action potential duration and upstroke velocity compared with CMs isolated from human EBs of similar age, but the maximal diastolic potential (MDP) was less negative [11]. This depolarized MDP may explain the improved excitation threshold and maximum capture rate observed in another study, where MDP was not reported [101]. The conduction velocity can be significantly higher in tissue constructs as opposed to two-dimensional monolayers [12], and in one report even approaches the values of native human heart [101]. The improved electrical conduction is probably due to improved cell alignment with good connectivity more analogous to that found in adult tissue [11]. On the other hand, electrical function-related genes, such as GJA1 (Cx43), SCN5A (Na_v_1.5), KCNH2 (Kir2.1), and CACNA1C (Ca_v_1.2), do not seem to have enhanced expression in three-dimensional cultures [12].

### Electrical stimulation

Cardiomyocytes are constantly subjected to electrical signals *in vivo* that promote synchronous contractions, and electrical activity provides important instructive cues during growth and development of the heart. Although limited work has been published, current data suggest that electrical stimulation promotes aspects of hPSC-CM maturation. Chronic electrical pacing of hESC-CMs at 1 Hz for 2 weeks resulted in more mature cells characterized by better structured and organized myofilaments [15]. Electrophysiological maturation was also evident as cells showed suppressed spontaneous activity, hyperpolarized resting potential, increased intracellular calcium transients, and increased expression of resting ion channel (Kir2.1), calcium handling (CSQ2, junctin, triadin, SERCA), structural (Cav3, Amp2) and contractile (myosin heavy chain (MHC), myosin light chain (MLC)) proteins. In another study on hESC-CMs, electrical stimulation for 4 days produced cell elongation, increased action potential duration, increased calcium transients, increased expression of ion channel (HCN1, SCN5A, Kv4.3), calcium handling (SERCA), and contractile (MLC2v) genes, and decreased the expression of other ion channel genes (HCN3, KCNQ1, KCNH2) [102]. No change was found in maximum diastolic potential. In hiPSC-CMs engineered to form spontaneously beating EHTs, electrical field stimulation at 1.5 to 2 Hz for at least 10 days led to CMs with improved alignment, improved cross striations, an elongated shape, a higher cytoplasm-to-nucleus ratio, and improved force development [103]. In other species, biphasic pulse trains produced dose-dependent increases in β-MHC and troponin-T expression in differentiating mESCs [104]. In isolated NRVMs, electrical pacing produced periodic contraction and induced cell alignment and coupling, markedly improved ultrastructural organization, and increased amplitude of contraction of NRVMs seeded on collagen sponges [105]. Pacing has also been shown to modulate action potential duration, Kv4.3 expression, calcium handling (NCX) expression, and conduction velocity in NRVM monolayers [106], and it increased excitability and expression of Cx43 in NRVMs cultured with endothelial cells in a polyethylene glycol diacrylate gel [107]. In spontaneously beating EHTs composed of NRVMs, electrical stimulation led to a more physiologic rightward shift in the force response curve to external calcium, higher CM density in the center of the EHT, increased Cx43 expression, and improved sarcomere ultrastructure with regular M-bands [103]. While there is general agreement in the literature with respect to the maturation effects of electrical stimulation, studies with NRVMs suggest that the best that can be achieved is an age-matched native phenotype rather than the adult phenotype [108].

Mechanistically, electrical stimulation initiates and coordinates cellular contraction, which regulates cell and tissue structure and function during development [40]. The beneficial effects of electrical pacing are likely to be mediated through the activation of intrinsic forces associated with cellular contraction, in particular, those arising from dynamic loading conditions at focal adhesions, at fascia adherens and desmosomes, and along elements of the cytoskeleton. Increases in protein synthesis and accelerated cell growth occur in neonatal rat and adult feline CMs with electrical stimulation, and are prevented when contraction is inhibited either by the addition of calcium channel blockers or the contraction inhibitor BDM [84, 109]. Electrical pacing may even have a greater stimulatory effect than externally applied stretch under some conditions [84], suggesting that mechanisms other than mechanical forces alone may spur maturation. The opening of calcium channels with electrical stimulation causes cyclic intracellular calcium transients, which can regulate a host of intracellular signaling pathways [110]. A single, long duration (90 seconds) DC electric field pulse can increase intracellular reactive oxygen species (ROS) and augment cardiac differentiation of non-contracting hESCs [111]. In mouse, electric fields also can augment cardiac differentiation, activate ROS and produce broad transcriptome changes [104], including activation of the transcription factor nuclear factor kappa B [112].

Other investigations on animal CMs indicate that electrical stimulation may be a viable approach for effecting metabolism, hormonal signaling (also see next section), and CM recruitment. NRVMs electrically stimulated in the presence of a contraction inhibitor experience up-regulation of several genes involved in metabolism: *Bckdha*, encoding a keotacid dehydrogenase, *Cpt1b*, encoding carnitine palmitoyltransferase 1b, *Gpam*, encoding glycerol-3-phosphate acyltransferase, and *Hadh*, encoding hydroxyacyl-CoA dehydrogenase [113]. Further, electrically stimulated adult rat CMs exhibit an increase in the rate of GLUT4 exocytosis but no change in the rate of GLUT4 endocytosis when compared to unstimulated controls [114]. Electrical stimulation of NRVMs at 5 Hz results in increased expression of COX and Cyt *C* mRNA, which are associated with mitochondrial proliferation. These are preceded by up-regulation of the transcriptional activation factor genes *c*-*fos*, *c*-*jun*, *JunB*, and *NRF1* [115]. Finally, the improved function of chronically paced EHTs may be due to both increased recruitment of CMs that participate in coordinated contraction as well as improved function of single CMs [103].

### Hypertrophic signals and metabolic adaptations

Post-natal physiological growth is stimulated by increased workloads and biomechanical stress (that is, physical cues), which stimulate hypertrophic responses and metabolic responses in CMs that may also influence the developmental maturation of hPSC-CMs. These *in vivo* stresses can be sensed by stretch-sensitive ion channels or integrins linked with cytoskeletal proteins that activate a plethora of signaling cascades, often involving calcium. Some of these signaling cascades are likely intrinsic to hESC-CMs, because equiaxial cyclic stretch promotes an increase in cell size, cytoskeletal assembly and improved sarcomeric organization in two-dimensional cultures [116]. We have also observed that hPSC-CMs cultivated in serum produce larger cells (particularly with some iPSC lines) than those cultivated in fully defined, serum-free conditions. Stimulation of the p38-MAPK signaling cascade, which in rodent responds to serum, leads to an increase in cell size, improved sarcomere and cytoskeletal assembly, and physical cell traits like elongation that are consistent with more mature cells [116, 117]. Thyroid hormone, a known hypertrophic stimulatory hormone, increases CM size, sarcomere length, contractile forces and anisotropy of iPSC-CMs, while simultaneously reducing cell cycle activity [18]. Similarly, adrenergic agonists that affect cardiac function and growth like phenylephrine (α-adrenergic agonist) produce hypertrophy, while isoproterenol (β-adrenergic agonist) only affects chronotropic activity in two-dimensional cultures [116]. Mechanical stimulation coupled with delayed electrical activation, unlike electrical or mechanical stimulation alone, improved SERCA2a and TNNT2 expression in NRVM engineered tissues. These improvements were due in part to the increased expression and phosphorylation of AKT/PKB, an important downstream target for insulin-like growth factor 1 (IGF-1)/phosphoinositol-3-kinase mediated hypertrophic growth [118]. Consistent with a growth role for IGF-1 in these cells, Park and colleagues showed that the combined effects of IGF-1 and electrical stimulation can improve the overall contraction strength, sarcomere development and Cx43 expression of NRVM cardiac tissue constructs [119].

The heart undergoes significant metabolic changes during the perinatal period, only a few of which have been demonstrated in PSC-CMs. Undifferentiated PSCs from mouse and human depend on glycolysis, and mESCs have lower basal respiratory rates, lower maximal respiratory capacity, and increased glycolysis than mESC-CMs. The latter exhibits higher energetic requirements that necessitate some degree of oxidative metabolism [65]. When compared to fetal heart CMs, hESC-CMs differ in energy metabolic processes involving the Krebs cycle, cellular respiration, mitochondrial biogenesis, and lipid metabolism [59]. hiPSC-CMs rely principally on glycolysis when cultured in media containing glucose, even if present at low levels and when fatty acids are available [120]. hiPSC-CMs, however, can shift to oxidative phosphorylation for ATP generation when cultured in galactose-containing media. When cultured under these conditions or supplemented with fatty acids, hiPSC-CMs exhibit higher reserve and respiratory capacities, and more closely resemble the bioenergetics of adult CMs [120]. Whether the activated oxidative phosphorylation in galactose media represents a normal metabolic induction or a stress response remains unclear. By taking advantage of the fact that lactate is used in Krebs cycle metabolism by CMs but not by non-CMs, glucose-depleted and lactose-supplemented media enrich for hESC-CMs and hiPSC-CMs [121]. This effect can be attributed both to the fact that ESCs have a lower expression of genes encoding enzymes involved in the Krebs cycle and are unable to obtain large amounts of ATP by oxidative phosphorylation or by glycolysis under glucose-deprived conditions. These cells are unable to convert lactate to glucose-6-phosphate for use in the Krebs cycle because it requires the expenditure of ATP [121]. It is, however, unclear what types of CMs are isolated following lactate purification. Finally, use of a five factor cocktail to promote adipogenesis and fatty acid metabolism in ARVD/C mutant containing iPSC-CMs promoted manifestation of the disease phenotype *in vitro*. This phenotype could not be reproduced in standard cardiogenic conditions, illustrating how metabolism can be used to promote a more adult-like phenotype critical to study disease pathogenesis [83].

Although the effects of mitochondrial function and metabolism on the maintenance of pluripotency, as well as on PSC reprogramming and differentiation efficiencies, have been aptly covered by several reviews [122–124], a few points are worth mentioning. Differentiation of hESCs is characterized by increases in mitochondrial mass and DNA content, as well as an increase in ATP and ROS [125]. Further, mesodermal commitment can be affected by chemically induced changes in mitochondrial biogenesis [126]. Work in mESCs suggests that disruption of the mitochondrial respiratory chain during early differentiation of ESC-CMs not only compromises mitochondrial content, localization, and arrangement, but also disrupts sarcomere formation, resulting in a decreased yield of functional CMs [65]. In undifferentiated hESCs, mitochondria exhibit perinuclear organization and a rounded phenotype [126], while differentiation results in the development of branched mitochondria into an extensive network [127]. Thus, mitochondrial function may be critical to structural changes that occur in hESC-CMs in response to mechanical loads. Given the complexity of metabolic and mitochondrial adaptations that occur during development and *in vitro*, readers are directed to in-depth reviews of mitochondrial biogenesis for further information [128, 129].

## Future areas of investigation

To better understand the mechanisms of mechanical and electrical cues on developmental properties of PSC-CMs, a number of variables need to be considered. First, CMs *in vivo* do not function alone, and are normally in contact with fibroblasts, smooth muscle cells and other vascular cells. Kim and colleagues [130], for example, showed that purified hESC-CMs isolated from early EB cultures failed to develop adequate intracellular Ca^2+^ handling protein and ion channel functions associated with electrophysiological maturation. The addition of non-CMs to the purified cells, however, could rescue this developmental loss, presumably either through cell-cell contact or the release of paracrine factors. Moreover, three-dimensional cardiac tissues form best when co-cultured with multipotent stromal cells or fibroblasts [101], but their contributions to structural and mechanical stimulation are unknown. Second, in depth transcriptomic analyses should be considered. While microarrays have been performed on two-dimensional cultures of PSC-CMs and compared with those performed on fetal heart samples, no analysis has systematically compared PSC-CMs subjected to physical stimulation in both two and three dimensions. In the absence of targeted mechanical or electrical interventions, these analyses are likely to be difficult to interpret since both electrical and mechanical stimulation can affect signaling cascades, nuclear signaling, transcription, metabolism and remodeling. Identification of specific electromechanical sensitive signaling pathways will require experimental interventions to uncouple stress, strain, electrically regulated forces during adaptive phases of remodeling, and complex data set analyses to unravel mechanisms. Similar to what has been done for pluripotency with hPSCs, it is likely that transcriptomic profiles may be able to define developmental maturation stages of *in vitro* derived CMs [131]. Third, iPSCs derived from patients with mutations that affect mechanical and/or electrical properties of PSC-CMs are likely to be highly informative. *In vitro* studies using iPSCs derived from probands with ARVD/C, a disease of the desmosome, have already shown that the induction of adult-like metabolism is critical for the establishment of this disease [83]. Moreover, cells with mutations in HERG/KCNH2 channels that cause long QT syndrome 2 have prolonged action potential durations and other electrical abnormalities that may affect maturation processes [132, 133]. It is also possible that altered epigenetic states in iPSCs may respond differently to mechanical signals and electrical stimulation. Theoretically, iPSCs might contain residual epigenetic memory of the founder line (for example, fibroblasts or blood), which could affect cytoskeleton proteins and signal transduction through the cytoskeleton differently than in CMs derived from PSCs. Detailed studies of this process, however, suggest that epigenetic memory may be short-lived in high quality iPSC lines, and that it may depend on the tissue of origin [134, 135]. Fourth, stretch-sensitive ion channels represent a potentially informative line of investigation that has implications not only for physical cues like stretch, but also for hypertrophic signaling. Moreover, ionic current flow and voltage gradients could be the basis for long-range signaling that could coordinate tissue growth and function [136]. Downstream signaling events that may be activated in response to mechanical (for example, stretch-sensitive channels) and electrical stimuli (ion and voltage-dependent channels) must also be considered. Intrinsic to this analysis, the role of the cytoskeleton and the ECM will need to be emphasized. Ultimately, attempts must be made to understand how any activated signaling cascade interfaces with intrinsic or cell autonomous maturation pathways. Finally, the identification of reference markers to assess heterogeneity and cell maturation will be critical to future studies designed to understand the responses of hPSC-CMs to stimuli. Although often ignored, human PSC-CMs are highly heterogeneous and can consist of multiple cell types. The ventricular, atrial and nodal cell types typically generated with *in vitro* differentiation also display different 'maturation’ states. Ultimately, improved surface markers will need to be identified that will permit the use of flow cytometry to assess heterogeneity and cell quality, as well as the isolation of sub-populations of hPSC-CMs with known traits. Although markers like VCAM1 and SIRPA have been identified as useful for isolating hPSC-CMs, these markers do not distinguish among subtypes of maturation states [137, 138]. Accordingly, optimized staining and isolation protocols will be required to advance this field and further studies involving physical cues.

## Conclusion

Significant research efforts have been undertaken to improve the generation and quality of hESC and hiPSC cell lines, and delineate mechanisms that promote CM commitment and differentiation. This has led to significant advances in our ability to routinely generate tens to hundreds of millions of hPSC-CMs for investigative or therapeutic applications. A major limitation to the use of these cells is their relative developmental immaturity. Human PSC-CMs are most similar to CMs obtained from embryonic or fetal hearts. Arrhythmic properties and weak contractile forces, in particular, pose confounding problems for disease replacement therapies; however, these same properties may prove valuable as models for drug screening, in terms of increased assay sensitivity. The *in vitro* differentiation system also has inherent deficiencies that may limit our ability to generate functional heart muscle. One of the major limitations is the lack of the normal organogenesis, of morphogens and growth factor gradients, and of blood circulation, all of which contribute to normal heart development and function. It therefore remains an open question as to whether terminal maturation of hPSC-CMs can be fully achieved *in vitro*. Post-transplantation of hPSC-CMs has, however, resulted in CMs with well-developed sarcomeric structures and morphologies similar to those found in adult heart. These latter results indicate that hPSC-CMs are fully committed and capable of forming functional heart muscle without normal organogenesis, but it remains unclear how similar they are to native myocardium.

The application of physical cues (electrical and mechanical) that occur during *in vivo* cardiac development may prove critical for maturation of hPSC-CMs *in vitro*. The goal would be to replicate cardiac perinatal development and to understand the mechanisms responsible for these adaptive changes. To achieve this goal, we submit that hPSC-CMs, and ultimately ventricular CMs with defined/known developmental stages, will need to be examined in complex three-dimensional tissue constructs that can be subjected to mechanical, electrical, hypertrophic and metabolic stimuli. Unlike traditional two-dimensional systems, these engineered constructs should allow for dynamic feedback between electro-mechanical signaling and ECM remodeling, as well as adaptive changes in cell and tissue architecture, analogous to what naturally occurs in the heart. Coupling advances in three-dimensional tissue design with physical cues should lead to the development of more natural cardiac tissues amenable to robust mechanistic analysis that have clinical relevance for modeling and eventually treating cardiac syndromes.

## Note

This article is part of a thematic series on *Cardiovascular regeneration* edited by Ronald Li. Other articles in the series can be found online at http://stemcellres.com/series/cardiovascular.

## Abbreviations

ARVD/C: Arrhythmogenic right ventricular dysplasia/cardiomyopathy
BDM: 2,3-butanedione monoxime
CM: Cardiomyocyte
Cx43: Connexin 43
EB: Embryoid body
ECM: Extracellular matrix
EHT: Engineered heart tissue
ESC: Embryonic stem cell
FAK: Focal adhesion kinase
hESC-CM: Human embryonic stem cell-derived cardiomyocyte
hiPSC-CM: Human induced pluripotent stem cell-derived cardiomyocyte
hPSC: Human pluripotent stem cell
hPSC-CM: Human pluripotent stem cell-derived cardiomyocyte
ICD: Intercalated disc
IGF-1: Insulin-like growth factor 1
iPSC: Induced pluripotent stem cell
MAPK: Mitogen-activated protein kinase
MDP: Maximal diastolic potential
mESC: Mouse embryonic stem cell
MLC: Myosin light chain
MYH: Myosin heavy chain
NRVM: Neonatal rat ventricular myocyte
PSC: Pluripotent stem cell
ROS: Reactive oxygen species
TNNI3: Cardiac troponin I
TNNT2: Cardiac troponin T.
